# Design Method of Freeform Surface Optical Systems with Low Coupling Position Error Sensitivity

**DOI:** 10.3390/s24134387

**Published:** 2024-07-06

**Authors:** Yu Xia, Yawei Huang, Changxiang Yan, Mingxiao Shao

**Affiliations:** 1 Changchun Institute of Optics, Fine Mechanics and Physics, Chinese Academy of Sciences, Changchun 130033, China; 18844181432@163.com (Y.X.);; 2 University of Chinese Academy of Sciences, Beijing 100049, China; 3 Center of Materials Science and Optoelectrics Engineering, University of Chinese Academy of Sciences, Beijing 100049, China

**Keywords:** freeform surface, desensitization, position error

## Abstract

Freeform off-axis reflective systems are significantly more difficult to align and assemble owing to their asymmetric surface shapes and system structures. In this study, a freeform surface system design method with low coupling position error sensitivity (FCPESM) was proposed. First, we established a mathematical model of a reflective system when it was perturbed by coupling position errors and used the clustering-microelement method to establish the coupling error sensitivity evaluation function. The evaluation function was then applied to the design process of a freeform surface off-axis three-mirror optical system. The results showed that the FCPESM optical design method can significantly relax the assembly tolerance requirements of optical systems on the basis of ensuring image performance. In this study, the reflective system was perturbed by tilt and decenter simultaneously, and the disturbance mechanism of position errors on optical systems was further improved. Through this research, freeform surface systems with both image performance and error sensitivity can be obtained, which makes freeform off-axis reflective systems with better engineering realizability.

## 1. Introduction

Reflective systems have the advantages of light weight, high transmission, radiation resistance, absence of chromatic aberrations, and thermal stability compared with refractive systems [1,2,3,4,5,6,7]. However, central obscuration in traditional coaxial reflective systems limits the resolution, energy concentration, and field of view (FOV). Therefore, off-axis reflective systems have been developed to eliminate obscuration and enlarge fields of view. The biased FOV and off-axis aperture will break the rotational symmetry of the optical system and induce many unconventional and asymmetric aberrations, such as field-constant coma and astigmatism, field-asymmetric astigmatism, and field-conjugate astigmatism, etc. It is difficult to correct these aberrations induced by asymmetry using traditional rotationally symmetric surfaces such as spherical and aspherical surfaces. Freeform surfaces have more degrees of freedom to reduce the asymmetric aberrations. With continuous improvement in application requirements, reflective optical systems are being developed in the direction of surface complexity [4,8,9,10,11,12,13,14,15,16,17,18,19].

The assembly of an off-axis system is difficult [1,20]. Since a freeform surface does not have an axis of rotational symmetry, the assembly of an off-axis system that contains freeform surfaces is generally more difficult than that comprising surfaces with revolution symmetry. The position error sensitivity indicates the effect of assembly errors on the imaging performance of optical systems. Reducing the position error sensitivity of optical systems can relax tolerance requirements, reduce the difficulty of processing and assembly, reduce the manufacturing cost of optical systems, and make optical systems more feasible [4]. Therefore, the design of freeform surface reflective systems with low position error sensitivity is of great significance for reducing the requirement of manufacturing accuracy and expanding the application range of freeform surfaces [21,22,23].

The direct optimization method is one of the earliest methods for the desensitization design of optical systems, which belongs to qualitative design method [24,25,26,27]. In the design process, the method does not explore the characteristic parameters of optical systems that have a deep mathematical relationship with error sensitivity but only needs to determine the conditional factors that have a high correlation trend with the error sensitivity of optical systems. By controlling these conditional factors, an optical system with low error sensitivity can be obtained. In 2003, Fuse established a multistructured optical system that contained a variety of positive and negative error forms. When all structures are optimized to obtain satisfactory results, the error sensitivity optimization of the optical system can be considered complete. In 2018, Liu et al. used this method to obtain an off-axis three-mirror optical system with low error sensitivity based on the concept of construction iteration [3]. In 2021, Carrión-Higueras obtained a high-order aspheric microlens with low error sensitivity by reducing the number of high-order aspheric coefficients and establishing an optimized multiple structure [27]. However, owing to the lack of quantitative characterization indicators and judgment criteria, it is often necessary to optimize a large number of samples and analyze the tolerance of a large number of optimization results. This design process is not only inefficient but also blind.

In view of the above problems, scholars have conducted research on the quantitative evaluation of the error sensitivity of optical systems. Many scholars have pointed out that the error sensitivity of optical systems is essentially the degree of error damage to the aberration field balance, and that there is a strong correlation between the error sensitivity of optical systems and aberration [28]. The aberration control method indicates that the error sensitivity can be reduced by optimizing the magnitude of aberrations of an optical system before and after an error disturbance [24,29,30,31]. In 2021, Lirong Wang proposed CS and AS evaluation functions. The expressions CS and AS for the sensitivity of uniform coma and linear astigmatism to surface tilt can be obtained by ignoring the constant term and 2nd order infinitesimal change in the coma and astigmatism Seidel aberration coefficients [32]. In 2021, Liu et al. proposed a lower aberration compensation design method to realize the design of a small infrared micro-optical system [30]. In 2022, Sasian proposed a method to reduce the error sensitivity by controlling the uniform coma and linear astigmatism of optical systems [29]. The above studies have proven that there is a close connection between the error sensitivity of optical systems and aberrations; however, a definite quantitative relationship has not been obtained.

The parameter control method refers to exploring the deep relationship between certain parameters and error sensitivity, and establishing a low error sensitivity design method centered on parameter control [2,33,34,35]. In 2021, Qin et al. reduced the tilt error sensitivity by controlling the slope of the mirror at the intersection of the incident ray and the mirror. With the same tilt error perturbation, the tilt error sensitivity of the optical system designed using this method was 53.8% of that of the initial system [36]. In 2023, Qin et al. deduced the relationship between the optical path variation and optical system parameters when the optical system is perturbed by a single position error and established a low error sensitivity optical design method (LC) centered on curvature control. The average error sensitivity value of the optical system optimized by this method was 50% of the original value under the same error conditions [37]. Earlier research results have demonstrated that it is feasible to quantitatively assess the error sensitivity of optical systems by exploring the deep relationship between certain parameters and the error sensitivity.

These studies provide different schemes for the desensitization design of optical systems; however, there are still some deficiencies. At present, there are few studies on the design of freeform surface desensitization. Freeform surface desensitization design methods can be divided into two categories: one is the qualitative design method, which does not explore the principle, but only focuses on the results. In order to obtain ideal optimization results, tolerance analysis and as-built performance evaluation on considerable systems must be carried out, which consumes a lot of calculation and optimization time [37]. The other is the quantitative design method, which can deeply explore the disturbance mechanism of the position errors to optical systems. However, the current research on freeform surfaces is aimed at the influence of the single position error on optical systems. In practical working situations, optical systems are often perturbed by multiple forms of errors at the same time [38].

In order to design freeform surface optical systems with low coupling error sensitivity, we carried out the following work. In Section 2, we established a mathematical model of the reflective system when it was perturbed by the coupling position error and used the clustering-microelement method to establish the coupling error sensitivity evaluation function of freeform surface systems. In Section 3, a freeform surface system design method with low coupling position error sensitivity was proposed (FCPESM). This method was then used to design a freeform off-axis three-mirror optical system. The design operates at F/4 with a 1000 mm focal length and a 2° × 2° FOV. Under the same constraints, we also designed an optical system using the single error sensitivity optimization method (LC). The results of the coupling position error sensitivity analysis demonstrated the effectiveness and superiority of the proposed method. The study in this paper can greatly relax the tolerance requirements in the assembly process of freeform surface systems and further reduce the difficulty of processing and assembly of freeform surface systems.

## 2. Theoretical Analysis of Coupling Position Error Sensitivity

### 2.1. Mathematical Model of Coupling Position Error Sensitivity

When the optical system is perturbed by errors such as tilt, decenter, etc., the balance of the optical system will be disrupted. Each imaging ray will produce optical path variation caused by spatial position misalignment [36]. The smaller the optical path variation (*OPV*), the closer the system is to the state before perturbation, so the value of *OPV* can be used as the evaluation standard of the error sensitivity of optical systems.

Both tilt and decenter errors are important manifestations of the spatial position misalignment of optical components. Tilt refers to the rotation angle value centered on the vertex of the optical surface about the tangential or sagittal axis, which we set as α. Decenter refers to the distance that the optical element translates in the tangential or sagittal direction with the surface vertex as its center, which we set as h. Many scholars have studied the situation of tilt error or decenter error acting alone on the surface of optical components; however, they often exist in optical systems simultaneously in the form of coupling position errors. In this section, ray tracing is applied to analyze the mathematical relationship between the *OPV* and the parameters of the optical system when the optical system is interrupted by coupling position errors.

**Figure 1:**
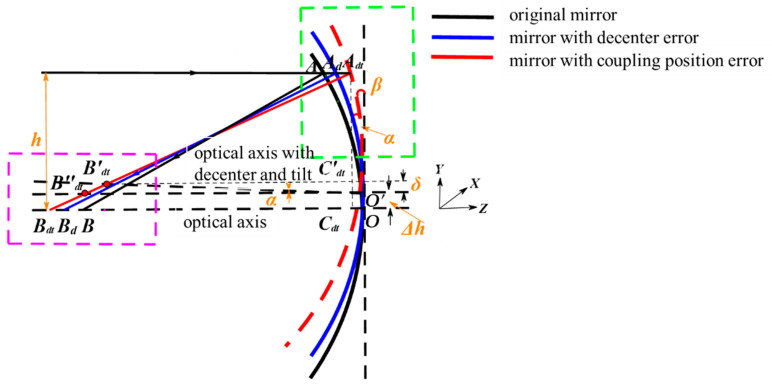
Coupling error sensitivity mathematical analysis model.

Figure 1 presents a mathematical model of the reflective optical surface after the coupling position error perturbation. In the figure, the *Z*-axis is the optical axis, and the incident ray propagates in the direction of the optical axis. The black curve represents the original mirror, and the center of the mirror intersects the optical axis at point *O*. The blue curve represents the mirror with the decenter error, and the red curve represents the mirror with the coupling position error. The incident ray intersects the original mirror at point *A*, and the reflective ray intersects the optical axis at point *B*. When the decenter error is applied to the mirror, the value of the decenter can be expressed as ∆*h*. The incident ray intersects the mirror at $A_{d}$,and the reflective ray intersects the optical axis at $B_{d}$. When the mirror is applied a tilt error on the basis of being applied the decenter error, the tilt angle is α. The incident ray intersects the mirror at $A_{dt}$, and the reflective ray intersects the optical axis at point $B_{dt}$.

When the optical system is perturbed by coupling position errors, the *OPV* can be expressed as

(1) $$OPV=AA_{dt}+A_{dt}B_{dt}-AB,$$

where the unit of *OPV* is measured in millimeters, and all distance units in this study are expressed in millimeters.

We select the quadratic surface as the mirror base. Using the center of the mirror point *O* as the coordinate origin to establish the Cartesian coordinate system, the mirror equation can be expressed as follows:

(2) $$z=\frac{cr^{2}}{1+\sqrt{1-(k+1)c^{2}r^{2}}},$$

where *z* is the sag of the surface parallel to the optical axis, *c* (mm^−1^) is the curvature of the surface, and *r* is the radial distance.

We select the *YOZ* plane for analysis, and the mirror equation can be simplified as

(3) $$z=\frac{cy^{2}}{1+\sqrt{1-(k+1)c^{2}y^{2}}},$$

as shown in Figure 1, *c* < 0. We can obtain the coordinates of point *A* and point *B* as (*h*, $\frac{ch^{2}}{1+\sqrt{1-(k+1)c^{2}h^{2}}}$) and (0,−*L*), respectively, where *h* is the height of the incident ray and *L* is the distance from the image plane to the mirror. The length of *AB* can be calculated as

(4) $$AB=\sqrt{h^{2}+{\left(L+\frac{ch^{2}}{1+\sqrt{1-(k+1)c^{2}h^{2}}}\right)}^{2}},$$

according to the amount of decenter error ∆*h*, the mirror expression after applying the error can be obtained as

(5) $$z=\frac{c{\left(y-\Delta h\right)}^{2}}{1+\sqrt{1-(k+1)c^{2}{\left(y-\Delta h\right)}^{2}}}.$$

In the green box in Figure 1, the length of $A_{d}A_{dt}$ can be calculated as

(6) $$\begin{array}{ll} A_{d}A_{dt} & =(h-\Delta h)\tan(\alpha +\beta )-(h-\Delta h)\tan\beta \\ & \approx (h-\Delta h)\tan\alpha \end{array}.$$

The length of $AA_{dt}$ can be calculated as

(7) $$\begin{array}{ll} AA_{dt} & =AA_{d}+A_{d}A_{dt}\\ & =\frac{c{\left(h-\Delta h\right)}^{2}}{1+\sqrt{1-(k+1)c^{2}{\left(h-\Delta h\right)}^{2}}}-\frac{ch^{2}}{1+\sqrt{1-(k+1)c^{2}h^{2}}}+(h-\Delta h)\tan\alpha \end{array}.$$

Make the optical axis containing the decenter error through the point *O*′ and draw an optical axis with a tilt angle of α. $A_{dt}B_{dt}$ intersects the tilt optical axis at the point $B\prime_{dt}$ and intersects the decenter optical axis at the point $B\prime \prime_{dt}$. Make a vertical line through the point $A_{dt}$ to the optical axis to intersect the original optical axis at the point $C_{dt}$ and make a line parallel to the optical axis through the point $B\prime_{dt}$ to intersect the line $A_{dt}C_{dt}$ at the point $C\prime_{dt}$. From the properties of similar triangles, it follows that

(8) $$\frac{A_{dt}B_{dt}^{\prime }}{h-\Delta h-L\sin\alpha }=\frac{A_{dt}B_{dt}}{h}.$$

In the purple box in Figure 1, $A_{dt}B_{dt}^{\prime }$ can be obtained by the following equation:

(9) $$A_{dt}B_{dt}^{\prime }=\sqrt{{\left(h-\Delta h-L\sin\alpha \right)}^{2}+{\left(L\cos\alpha +\frac{c{\left(h-\Delta h\right)}^{2}}{1+\sqrt{1-(k+1)c^{2}{\left(h-\Delta h\right)}^{2}}}+(h-\Delta h)\tan\alpha \right)}^{2}}.$$

Therefore, the variation of optical path after coupling error disturbance can be calculated as

(10) $$\begin{array}{ll} OPV & =AA_{dt}+A_{dt}B_{dt}-AB\\ & =\frac{c{\left(h-\Delta h\right)}^{2}}{1+\sqrt{1-(k+1)c^{2}{\left(h-\Delta h\right)}^{2}}}-\frac{ch^{2}}{1+\sqrt{1-(k+1)c^{2}h^{2}}}+(h-\Delta h)\tan\alpha +\frac{h}{h-\Delta h-L\sin\alpha }\cdot \\ & \sqrt{{\left(h-\Delta h-L\sin\alpha \right)}^{2}+{\left(L\cos\alpha +\frac{c{\left(h-\Delta h\right)}^{2}}{1+\sqrt{1-(k+1)c^{2}{\left(h-\Delta h\right)}^{2}}}+(h-\Delta h)\tan\alpha \right)}^{2}}\\ & -\sqrt{h^{2}+{\left(L+\frac{ch^{2}}{1+\sqrt{1-(k+1)c^{2}h^{2}}}\right)}^{2}} \end{array}.$$

We take the ellipsoidal surface as an example to explain Equation (10). The quadratic surface coefficient k=−1. We select the *YOZ* plane for analysis, and the mirror equation can be simplified as

(11) $$z=\frac{1}{2}cy^{2}(z<0).$$

According to the amount of decenter error ∆*h*, the mirror expression after applying the error can be obtained as

(12) $$z=\frac{1}{2}c{\left(y-\Delta h\right)}^{2}(z<0).$$

The variation of optical path after coupling error disturbance can be calculated as

(13) $$\begin{array}{ll} OPV & =AA_{dt}+A_{dt}B_{dt}-AB\\ & =c\Delta h(\frac{1}{2}\Delta h-h)+(h-\Delta h)\tan\alpha +\frac{h}{h-\Delta h-L\sin\alpha }\cdot \\ & \sqrt{{\left(h-\Delta h-L\sin\alpha \right)}^{2}+{\left(L\cos\alpha +\frac{1}{2}c{\left(h-\Delta h\right)}^{2}+(h-\Delta h)\tan\alpha \right)}^{2}}\\ & -\sqrt{h^{2}+{\left(L+\frac{1}{2}ch^{2}\right)}^{2}} \end{array}.$$

**Figure 2:**
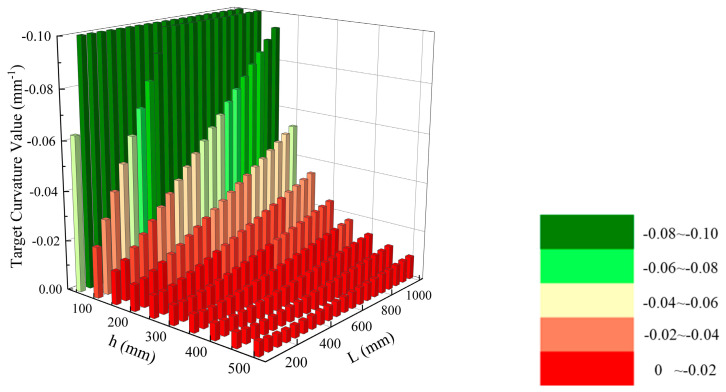
Distribution of target curvature values.

Different from the tilt error or the decenter error acting alone on the optical surface, when the incident height *h* and the distance *L* from the image plane to the mirror are fixed, the *OPV* no longer has a simple increasing or decreasing relationship with the curvature *c* of the mirror, but still has a clear analytical relationship. Equation (10) can be used to calculate the target curvature value for each target surface. We take the ellipsoidal surface as an example, when the tolerance of the optical system is assigned as follows: tilt tolerance of ±0.01° and decenter tolerance of ±0.01 mm, and the target curvature value of the optical system can be shown in Figure 2. Different combinations of *h* and *L* indicate that the target surface has different target curvature values, and the value of *OPV* is minimized at the target curvature value. In Figure 2, the range of target curvature values is distinguished using different colors.

### 2.2. Error Sensitivity Evaluation Function

**Figure 3:**
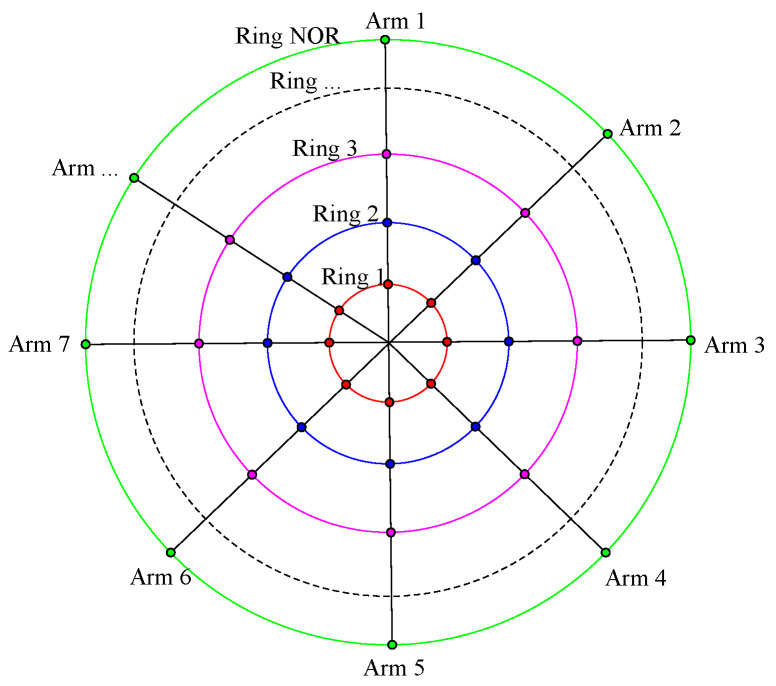
Location selection of sampling points.

Freeform surfaces do not have rotational symmetry; therefore, this study uses the microelement method (“Ring-Arm” pupil sampling method) to divide a freeform surface into several small regions. Each region can be regarded as a microquadric. There may be several target curvature values for the same target surface, as analyzed in Section 2.1, and each small region can be divided into N clusters based on the target curvature values. Reducing the error sensitivity of each cluster can lead to an optimized design for desensitization of the overall optical system. The sampling method is illustrated in Figure 3.

Next, the radius of curvature is used to establish the coupling position error sensitivity evaluation function of optical systems based on the idea of clustering-microelement. The coupling position error sensitivity of a cluster for an FOV point on a freeform surface is expressed as

(14) $$RC=\frac{1}{\sqrt{\sum_{u=1}^{U}U\cdot {\left(RC_{u}-RC_{0,u}\right)}^{2}}},$$

where *U* represents the number of sampling points, *u* represents the sequence number of sampling points. $RC_{0,u}$ represents the radius of curvature of a sampling region of the initial system.

The coupling position error sensitivity for all FOV points in a cluster is expressed as

(15) $$RC_{S,F}=\sqrt{\sum_{s}^{NOS}\sum_{f}^{NOF}\frac{RC_{s,f}^{2}}{NOS\cdot NOF}},$$

where *NOS* is the number of the surface, *s* is the serial number of the surface, *NOF* is the number of the FOV point, and *f* is the serial number of the FOV point.

The total position coupling error sensitivity of the optical system is expressed as

(16) $$RC_{cluster}=\frac{\sum_{i=1}^{N}RC_{S,F,i}}{N}.$$

## 3. Simulation and Verification of Desensitization Design Method

### 3.1. Desensitization Design Method

Based on the analysis in Section 2.1, we proposed a freeform surface system design method with low coupling position error sensitivity (FCPESM). The method can be divided into the following three steps:

(1) Construction of the initial freeform surface system according to the requirements of the optical system index.
(2) Establishment of coupling position error sensitivity evaluation function. First, the microelement method is used to divide the freeform surface, and the target curvature value of each microquadric can be calculated according to Equation (10). Then, the system coupling position error sensitivity evaluation function can be constructed based on the idea of clustering and Equation (14)~(16).
(3) Error sensitivity optimization. The optimization goal is to reduce the error sensitivity evaluation function value of the system. If the optical system meets the image performance requirements, the optical system needs to be continued to be optimized. If not, the system can be output as the final result.

### 3.2. Simulation of Desensitization Design Method

In this section, we provided an example of the technical support for the FCPESM method presented in Section 3.1. A freeform surface off-axis three-mirror optical system with a focal length of 1000 mm, F-number of 4, FOV of 2° × 2°, and an image performance evaluation wavelength of 588 nm was used as an example to explain the FCPESM method. The FOV in the sagittal direction is 9°~11° and the FOV in the tangential direction ranges from 0° to 2°. All optimizations in this study used the same Dell computer with Intel’s i7-10510U CPU @ 1.80GHz, which was made in China.

Similar to most design methods, the initial configuration of the optical system was established at the beginning. An off-axis three-mirror optical system can be obtained by biasing the aperture or field of view of coaxial systems or by searching from a library of initial structures built into existing optical design software. The second method was adopted in this study. The non-relayed COOK TMA system was selected as the optical system configuration, which was close to the design index of the target optical system.

**Figure 4:**
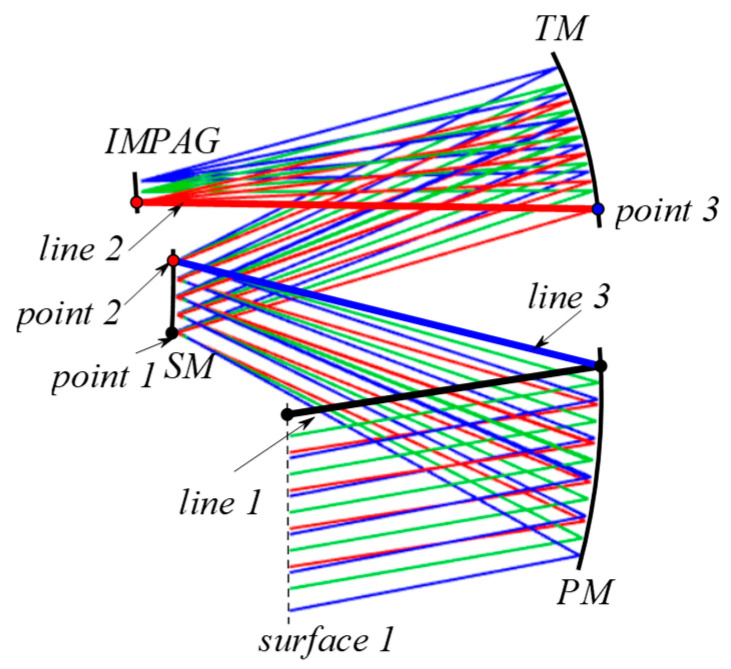
Ray-tracing constraints condition.

The configuration constraint conditions should be set before optimization. The constraint condition mainly includes system size restrictions and mirror intervention caused by mirror or ray crossing, which is significant for an off-axis three-mirror optical system. As shown in Figure 4, the PM, SM and TM are the primary, secondary, and triple mirrors of the off-axis three-mirror optical system, respectively. We can constrain several distance relationships to constrain the system size and avoid generating mirror intervention: the distance from the lower edge point of SM to the line 1, where line 1 is the line between the upper edge point of surface 1 and the upper edge point of PM; the distance from the upper edge point of SM to the line 2, where line 2 is the line between the lower edge point of TM and the lower edge point of IMAGE; the distance from the lower edge point of TM to the line 3, where line 3 is the line between the upper edge point of SM and the upper edge point of PM.

At the same time, to avoid the influence of the mirror magnification on the error sensitivity, the optical focal power distribution of each mirror was basically kept constant during the design process. Then, the merit function wavefront error (WFE) of image performance was decided according to the requirements. Usually, WFE is chosen as the merit function; other criteria, such as the MTF or PSF, can also be chosen.

**Figure 5:**
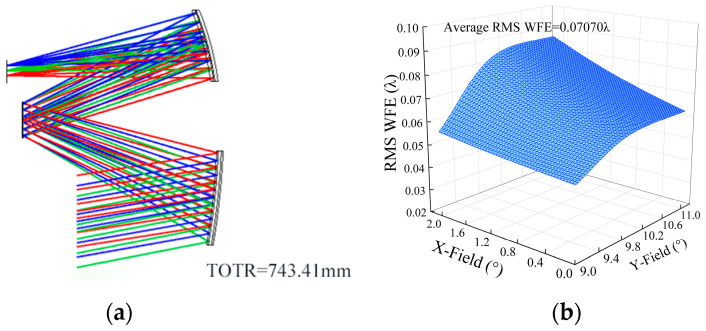
(**a**) “System 1” layout diagram; (**b**) “System 1” RMS WFE diagram.

After setting up the configuration constraints and selecting the evaluation function, we set the variables to start the optimization. In this example, the surface type of the three mirrors was upgraded to *xy* polynomial freeform surface in the order of TM, PM, and SM., using the first to sixth order terms. Then, the radius of curvature, thickness, quadratic surface coefficients, and freeform higher term coefficients of the three mirrors were set as variables. The system after image performance optimization was named “System 1”. The structural layout of “System 1” is shown in Figure 5a. The root mean square of the wavefront error RMS WFE of “System 1” is 0.07070λ, and the full field-of-view wavefront error are shown in Figure 5b. The surface parameters of “System 1” are listed in Table 1.

We selected 20 FOV points as the sampling points in a square FOV of 2° × 2°. The pupil sampling method corresponding to each FOV point is 3 Rings and 12 Arms, which means that one pupil is divided into 36 small areas. The target curvature radius value of each microquadric was calculated by Equation (10), and the microquadrics were classified into two clusters according to the inherent curvature radius value and the target curvature radius value of each microquadric. The clustering results are as follows: “Cluster 1” represents the microquadric that can reduce the coupling position error sensitivity by reducing the curvature radius value. This view is contrary to the conclusion obtained when the system is disturbed by a single position error; “Cluster 2” represents the microquadric that can reduce the coupling position error sensitivity by increasing the curvature radius value.

Bringing each sampling point and cluster into the coupling position error sensitivity evaluation function, the error sensitivity evaluation function applicable to this example was obtained, which can be expressed as

(17) $$RC=\frac{1}{\sqrt{\sum_{u=1}^{720}720\cdot {\left(RC_{u}-RC_{0,u}\right)}^{2}}},$$

(18) $$RC_{S,F}=\sqrt{\sum_{s=1}^{3}\sum_{f=1}^{20}\frac{RC_{s,f}^{2}}{3\cdot 20}},$$

(19) $$RC_{cluster}=\frac{\sum_{i=1}^{2}RC_{S,F,i}}{2}.$$

**Figure 6:**
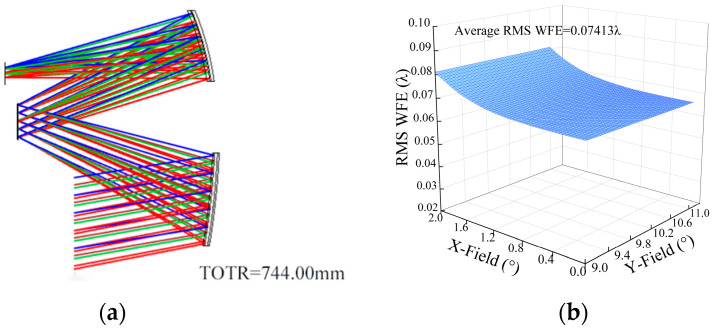
(**a**) “System 14” layout diagram; (**b**) “System 14” RMS WFE diagram.

The above process can be realized by writing macrofunctions using the Zemax software. The evaluation function value of “System 1” was calculated, $RC_{cluster}=2.3678$. Subsequently, the coupling position error sensitivity evaluation function was used for the desensitization design. The allowable image performance range of “System 1” was set as 0.07070λ ± 5%, and the maximum root mean square of wavefront error (RMS WFE) for 20 fields of view was less than 1/10λ. The optimization target was set as a $RC_{cluster}$ value less than 2.3678, and desensitization optimization was continued until the optical system image performance did not meet the requirements. The $RC_{cluster}$ value was continuously reduced through multiple optimizations to obtain “System 2”, “System 3”, …, “System 10” nine systems in total. The RMS WFE of “System 10” was 0.07426λ, the $RC_{cluster}$ value was 0.9133, and the imaging performance was just enough to satisfy the requirements. Keeping the $RC_{cluster}$ value of “System 10” unchanged, we obtained “System 11”, “System 12” … “System 16” six systems through optimization. Taking into account the image performance and coupling position error sensitivity, “System 14” was considered to be the optimal system. The structural layout of “System 14” is shown in Figure 6a. The RMS WFE of “System 14” is 0.07413λ, and the full field-of-view wavefront error is shown in Figure 6b. The surface parameters of “System 14” are listed in Table 2.

**Figure 7:**
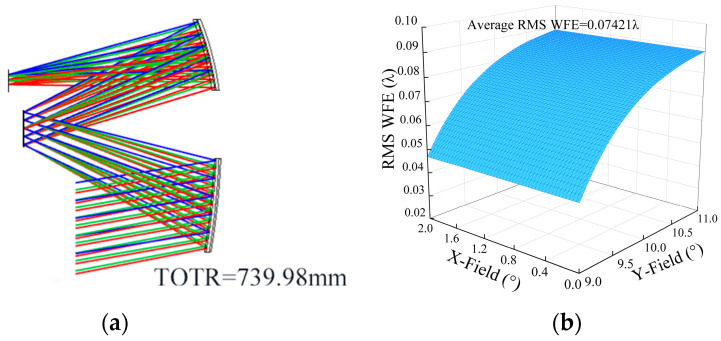
(**a**) “System 17” layout diagram; (**b**) “System 17” RMS WFE diagram.

In order to prove the effectiveness of the low coupling position error sensitivity optimization method (FCPESM) proposed in this study, the single error sensitivity optimization method (LC) was used for desensitization design as a comparison. The image performance constraints remained the same, and “System 17” was obtained by taking LC = 0.209 as the starting point and decreasing to LC = 0.194. The structural layout of “System 17” is shown in Figure 7a. The RMS WFE of “System 17” is 0.07421λ, and the full field-of-view wavefront error is shown in Figure 7b. The surface parameters of “System 17” are listed in Table 3.

### 3.3. Verification of Desensitization Design Method

**Figure 8:**
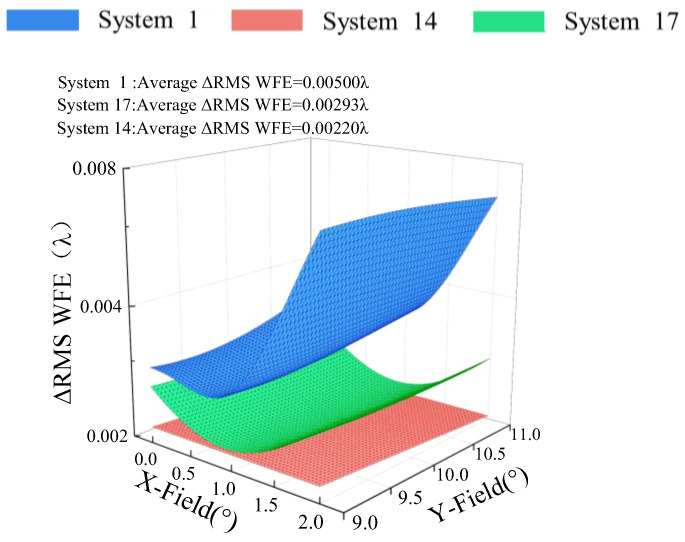
Full field ΔRMS comparison diagram.

- In order to verify the correctness and effectiveness of the FCPESM method, we conducted coupling error sensitivity analysis on “System 1”, “System 14” and “System 17”, respectively. The tolerance of the system was set to tilt ± 0.01°, decenter ± 0.01 mm. We used the Monte Carlo tolerance analysis method with 2000 samples to calculate the error sensitivity of the optical systems, with ΔRMS WFE as the error sensitivity evaluation criterion. ΔRMS WFE is the change in the RMS WFE before and after the error perturbation.
- The coupling position error sensitivity ΔRMS WFE comparison of the full field of view of “System 1”, “System 14” and “System 17” is shown in Figure 8. It can be seen from Figure 8 that after perturbation by the coupling error, the average ΔRMS WFE of “System 1” is 0.00500λ and the average ΔRMS WFE of “System 17” is 0.00293λ. With the same coupling position error perturbation, the average ΔRMS WFE of “System 14” is 0.00220λ. The average ΔRMS WFE of “System 14” obtained using the FCPESM method was reduced by 56% compared with that of the optical system before desensitization. The coupling position error sensitivity of “System 14” was reduced by 25% compared with “System 17”, proving the effectiveness of the FCPESM method.

**Figure 9:**
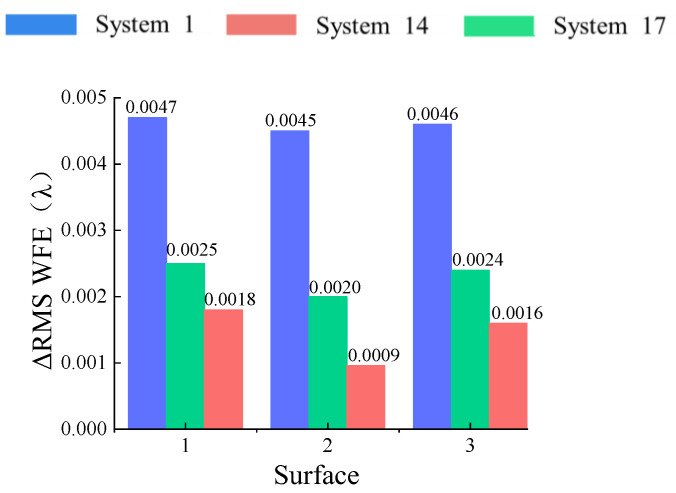
ΔRMS comparison of three optical surfaces.

The coupling position error sensitivity ΔRMS WFE comparison of each optical surface of “System 1”, “System 14” and “System 17” is shown in Figure 9. For surface 1, the ΔRMS WFE values of “System 1”, “System 17” and “System 14” are 0.0047λ, 0.0025λ and 0.0018λ, respectively, after being perturbed by the coupling error. For surface 2, the ΔRMS WFE values of “System 1”, “System 17” and “System 14” are 0.0045λ, 0.0020λ and 0.0009λ, respectively, after being perturbed by the coupling error. For surface 3, the ΔRMS WFE values of “System 1”, “System 17” and “System 14” are 0.0046λ, 0.0024λ and 0.0016λ, respectively, after being perturbed by the coupling error.

## 4. Conclusions

In this study, a novel low error sensitivity design method for freeform surface reflective systems is proposed to relax the assembly tolerance requirements and reduce the assembly difficulty of freeform surface systems. First, we established a mathematical model of the reflective system when it was perturbed by the coupling position error and used the clustering-microelement method to establish the coupling error sensitivity evaluation function of freeform surface systems. Then, by applying this evaluation function to the design of freeform off-axis reflective systems, a new desensitization design method for freeform reflective systems can be obtained. We designed two freeform surface off-axis three-mirror optical systems using the low coupling position error sensitivity optimization method (FCPESM) and the single error sensitivity optimization method (LC), respectively.

The results showed that the coupling error sensitivity obtained by the FCPESM method was reduced by 56% compared with that obtained by the traditional optical design method and was reduced by 25% compared with that obtained by the LC method within the permissible range of imaging performance. The FCPESM method can consider both the image performance and position error sensitivity of freeform surface systems. In this model the reflective system is perturbed by tilt and decenter simultaneously, which makes the position error sensitivity evaluation method of freeform surfaces more comprehensive. This design method can significantly reduce the disturbance of position errors on freeform surface systems. However, the FCPESM method only investigates the influence mechanism of position errors and does not consider surface figure errors or other types of error forms. In addition, the optical system designed using the FCPESM method has a narrow field of view. In the future, we intend to apply this method to large field-of-view optical systems and to investigate other types of errors so that the method can be better applied in engineering practice.

## Figures and Tables

**Table 1:** “System 1” surface parameters.

	Surface Type	Radius (mm)	Thickness (mm)	Conic	Order	*x* Tilt (mm)	*y* Decenter (mm)
PM	*xy* polynomial	−2496.633	−685.48	−2.40	6	4.74	−149.67
SM	*xy* polynomial	−740.014	675.71	2.99	6	−0.10	−0.39
TM	*xy* polynomial	−1034.194	−733.64	0.28	6	−11.59	143.49

**Table 2:** “System 14” surface parameters.

	Surface Type	Radius (mm)	Thickness (mm)	Conic	Order	*x* Tilt (mm)	*y* Decenter (mm)
PM	*xy* polynomial	−2527.665	−694.32	−2.49	6	4.80	−151.69
SM	*xy* polynomial	−747.80	685.22	−5.06	6	−0.11	0.178
TM	*xy* polynomial	−1038.61	−734.90	0.28	6	−11.80	147.62

**Table 3:** “System 17” surface parameters.

	Surface Type	Radius (mm)	Thickness (mm)	Conic	Order	*x* Tilt (mm)	*y* Decenter (mm)
PM	*xy* polynomial	−2490.58	−679.26	−2.51	6	3.38	−147.11
SM	*xy* polynomial	−744.74	681.32	−3.25	6	−0.087	−0.202
TM	*xy* polynomial	−1042.69	−739.98	0.29	6	−11.48	144.21

## Data Availability

Data underlying the results presented in this paper are not publicly available at this time but may be obtained from the authors upon reasonable request.
